# Surgical Technique for Revision of the Distally Migrated Fassier–Duval Femoral Rod in Osteogenesis Imperfecta: A Case Report

**DOI:** 10.3390/children12091269

**Published:** 2025-09-21

**Authors:** Peter Staunton, Pasin Tangadulrat, Reggie Charles Hamdy

**Affiliations:** 1 Department of Orthopaedics, University of Limerick Hospitals Group, V94 T9PX Limerick, Ireland; peter.staunton1@hse.ie; 2 Department of Orthopedics, Faculty of Medicine, Prince of Songkla University, Songkhla 90110, Thailand; pasin.t@psu.ac.th; 3 Pediatrics and Limb Deformity Unit, Department of Pediatric Orthopaedic Surgery, Shriners Hospital for Children, McGill University, Montreal, QC H4A 0A9, Canada; 4 Division of Pediatric Orthopaedic Surgery, Montreal Children’s Hospital, Montreal, QC H4A 3H9, Canada

**Keywords:** osteogenesis imperfecta, telescoping rod, Fassier–Duval rod, surgical technique, revision surgery, distal migration

## Abstract

**Highlights:**

**What are the main findings?**

- We describe a surgical technique for removing a distally migrated Fassier–Duval femoral rod in a patient with osteogenesis imperfecta
- The technique involves creating a retrieval channel retrogradely through an existing fracture or osteotomy site, allowing for the implant’s extraction while bypassing the critical proximal fixation area.

**What is the implication of the main finding?**

- This approach prioritizes the preservation of proximal femoral bone stock, which is crucial for achieving stable fixation of the subsequent revision rod.
- It provides surgeons with a clear, step-by-step strategy for a rare and challenging clinical problem not previously detailed in the literature, aiming to avoid difficult-to-salvage complications.

**Abstract:**

**Background/Objectives**: Managing long bone fractures and deformities in osteogenesis imperfecta (OI) with telescoping rods is a common but challenging procedure. A rare complication is the distal migration of the rod’s proximal female component, which complicates standard revision surgery. This article aims to describe a surgical technique for the revision of a distally migrated Fassier–Duval (FD) femoral rod. **Methods**: We present the case of an 8-year-old girl with OI type IV who experienced distal migration of her right femoral FD rod—the surgical technique involved extracting the rod retrogradely through the fracture/osteotomy site. We used a trephine to remove surrounding bone within the canal, thereby preserving the critical bone stock in the greater trochanter needed for secure fixation of the revision implant. **Results**: The distally migrated female component was successfully removed through the trephined canal with a combination of axial traction and rotational force. The proximal bone stock was preserved, allowing for the stable placement of a revision FD rod. **Conclusions**: The retrograde trephine technique is a viable and effective strategy for revising a distally migrated telescoping rod in patients with OI. This approach prioritizes the preservation of proximal bone stock, which is crucial for the stability and longevity of the revision implant.

## 1. Introduction

Managing long bone fractures and deformity in Osteogenesis Imperfecta (OI) is challenging. Growth and the tendency towards deformity and fracture require a comprehensive evaluation of surgical technique and implants used [1,2]. Telescoping rods are frequently used in this setting and may require multiple exchanges throughout a child’s lifetime [3]. The Fassier–Duval (FD) Telescopic Intra-Medullary System™ (OrthoPediatrics), provides one such option for the structural support of the growing long bone [4,5]. It is a telescoping rod by design, composed of a male and female component, allowing distraction between them. The male component is anchored in the distal femoral epiphysis, while the female component is anchored proximally in the cartilage of the greater trochanter. The threaded section of the female component should be anchored in the cartilaginous part of the greater trochanter and not proceed past the trochanteric growth plate to avoid potential migration of the female component over time. Avoiding breach of the trochanteric physis must be balanced against achieving adequate purchase in the cartilage to avoid fixation failure.

With growth, given appropriate proximal and distal fixation, the male component will continue to withdraw from the female component until the remaining growth exceeds the telescoping length, at which point they disengage. Deformity and bending of the rod may create friction between the male and female components, which can overcome the anchoring strength of either the proximal or distal fixation. Poor preparation of the female component when cutting it to length may also predispose it to high levels of friction between the male and female components, with subsequent deleterious effects.

Studies on FD rod failure have focused on the positioning of the male component in the distal epiphysis [6], with limited information available on the migration of the female component. We present a case of distal migration of the proximal fixation of the female component in a fractured femur, resulting in rod failure that required revision. When an exchange of such a rod is required, standard retrieval methods may not be applicable. It is important to note that the outer diameter of the proximal fixation is 7 mm for a 3.2 mm rod, 8.5 mm for a 4 mm rod, and 10 mm for a 4.8 mm rod, and so on. This reflects the size of access required for rod retrieval, meaning bone loss is inevitable. Considerations must be given to balancing retrieval morbidity against the remaining bone stock for fixation.

## 2. Materials and Methods

This study was undertaken as a case report. Therefore, per the institution’s policy, it was not formally supervised by an Institutional Review Board. Clinical information and radiographic images for the patient were obtained and reviewed retrospectively.

## 3. Results

**Figure 1:**
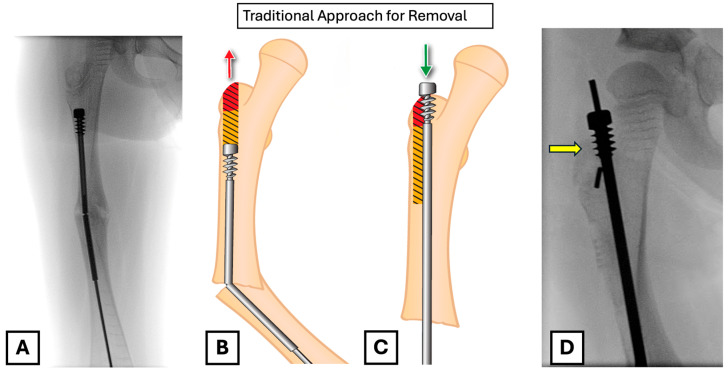
(**A**) Intra-operative femoral radiograph showing distal migration of the female component into the medullary canal, with associated rod failure and fracture. (**B**) Illustration of the predicted bone loss (yellow and red shaded area) with a traditional antegrade removal. This red area highlights the critical bone at the greater trochanter apophysis, which is required for secure fixation. (**C**) Illustration showing how antegrade removal compromises the fixation of a new FD rod, as the threads of the female component would be placed in an area of bone loss (red shaded). (**D**) Intra-operative radiograph showing the placement of the new FD rod; the female threads are in the red shaded area (yellow arrow), which will compromise a fixation with the traditional removal method.

We present the case of an 8-year-old girl with an underlying diagnosis of OI type IV. The diagnosis was confirmed with a genetic test showing a pathogenic heterozygous variant in the COL1A1 gene: c.994G > A (p.Gly332Arg), which results in the substitution of a glycine residue with arginine in the collagen chain. The patient has been treated and followed since the age of two. Her previous surgical history included bilateral tibial and femoral FD rodding. The patient had also been receiving yearly bisphosphonate therapy. The event of interest for this report is her right femur. Previously, the patient had the 3.2 mm FD rod implanted in her right femur for 4 years. During the follow-up period, the FD rod started to migrate distally, with its female component losing fixation from the greater trochanter apophysis (Figure 1A). The patient was otherwise asymptomatic, and the family did not want to undergo revision at that time. Eventually, a decision was made to revise the FD rod in her right femur when a new fracture occurred.

Given that the female component had migrated distally, we were left with two choices for retrieving it: antegrade through the greater trochanter or retrograde through the fracture. We recommend removing it retrogradely to prevent excessive bone loss in the proximal area, which could prevent solid fixation of the new implant. The anchoring of the revision rod in the greater trochanter is potentially compromised with the antegrade removal of this bone (Figure 1B,C).

## 4. Surgical Technique

### 4.1. Preparation

The patient is placed in the supine position on a radiolucent table following the administration of appropriate anesthesia. We typically utilize general anesthesia with an ipsilateral fascia-iliaca block. A bump is placed under the ipsilateral buttock to elevate the greater trochanter. The fluoroscopic C-arm is brought in from the opposite side, perpendicular to the table. Standard sterile preparation and draping are performed with the leg draped free, and exposure extending proximally to the costal margin. The level of the femur fracture or proposed osteotomy site is identified using fluoroscopy, and the corresponding incision for a lateral approach is marked on the skin.

### 4.2. Approach

**Figure 2:**
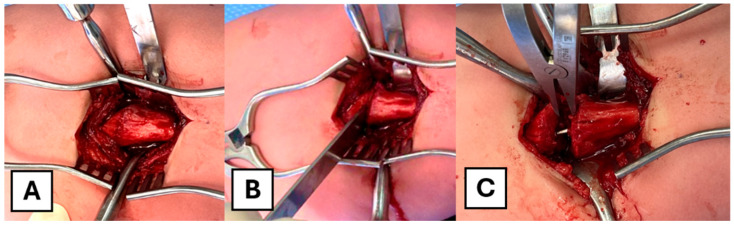
Intra-operative photographs of the surgical approach. (**A**) Exposure of the femur via a subvastus approach. (**B**) An osteotome is used to open the fracture site. (**C**) A laminar spreader is applied to distract the fracture site and expose the intramedullary rod.

A standard subvastus posterolateral approach to the femur is utilized. The skin is incised with a size 15 blade. A self-retainer is used to expose the subcutaneous fat, which is divided using cautery down to the level of the fascia. A Cobb elevator is used to sweep fat from the line of incision in the fascia, and it is incised longitudinally to the extent of the skin incision both proximally and distally. The periosteum is split, and the Cobb elevator is used to perform a sub-periosteal dissection. Broad Hohmann retractors are placed to expose the fracture/osteotomy site (Figure 2A). A straight osteotome is employed to open the fracture site (Figure 2B). The callus that is formed around the fracture site was removed to expose the fracture end. The fracture site/osteotomy site is then distracted with a laminar spreader. It is critical to distract slowly and gently, as forceful distraction, especially in a poor-quality bone or a more severe OI type, might lead to iatrogenic fracture. The underlying rod is then revealed. (Figure 2C).

### 4.3. Rod Retrieval

If the rod is intact, it should be cut at the fracture/osteotomy site with an appropriate device (e.g., wire cutter/metal cutting disk). By displacing the fracture ends, the distal male and female components can be extracted in an antegrade fashion. In this case, to retrieve the distally migrated female component, a bone nibbler or rongeur is first used to expose the component within the proximal segment. The guide wire was cannulated through the proximal female part to guide the trajectory and could be used for inserting a new FD rod later if the trajectory is optimal.

**Figure 3:**
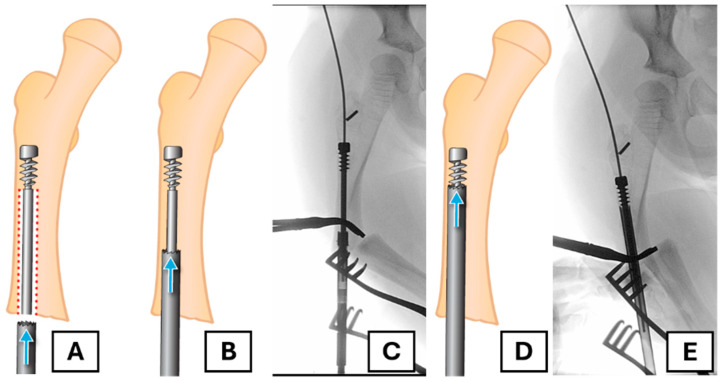
The retrograde trephine technique for implant removal. (**A**) Illustration of the trephine being placed over the migrated female component. The red dotted area shows the bone to be removed (**B**,**C**) The trephine advances over the rod, removing bone from within the canal, as shown in the illustration and corresponding radiograph. (**D**,**E**) Further advancement of the trephine towards the proximal fixation, confirmed by fluoroscopy.

To conserve as much bone as possible and avoid coring out the head of the female component, a retrograde approach is undertaken. A suitable trephine with an appropriate diameter (e.g., 7 mm for a 3.2 mm rod) and length is placed coaxially over the end of the female component. In this case, the FD trephine of an appropriate size was missing from the rescue set; therefore, we needed to use a separate trephine (7 mm inner diameter and a thickness of 1 mm). The surrounding bone is then removed along the extent of the rod as far as the proximal fixation (Figure 3A–E).

Using a trephine that passes over the female component provides more control and limits unnecessary bone loss, ensuring that the majority of bone loss occurs ideally within the canal. Prudent use of fluoroscopy is employed throughout this process to avoid unnecessary bone removal.

**Figure 4:**
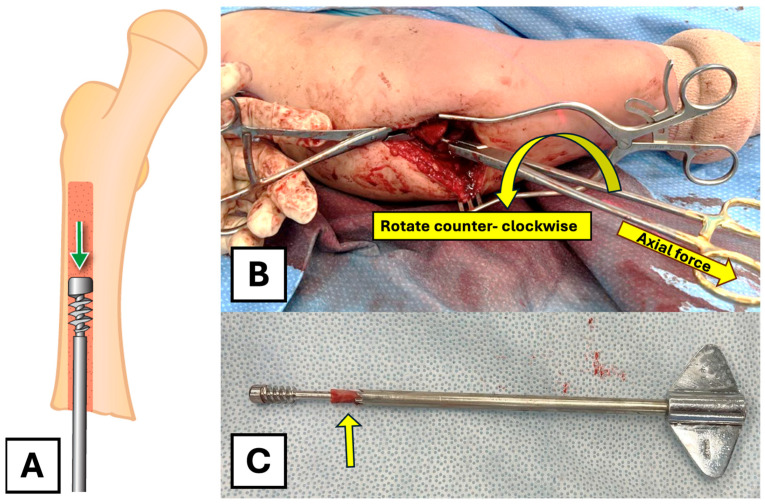
Extraction of the female component. (**A**) Illustration of the final extraction step after the path has been cleared. (**B**) A heavy wire-holding clamp is used to apply rotational and axial force to remove the female component through the fracture site. (**C**) The extracted female component, trephine, and the removed bone (arrow) were assembled to demonstrate the mechanism of the technique.

Following the removal of the trephine, there is room to pass a narrow, straight osteotome along the rod to circumferentially remove any remaining bony bridges between the trephined canal and the proximal fixation. Small, thin osteotomes can be used to free the head of the rod as needed from the surrounding bone. A heavy wire-holding clamp is then used to apply axial traction and rotational force to extract the female component (Figure 4A,B). Rotation in a counter-clockwise fashion is important to allow the threads of the proximal fixation to disengage from the surrounding bone. The removed female component and the trephine can be seen post-removal (Figure 4C).

### 4.4. Revision Rod Placement

**Figure 5:**
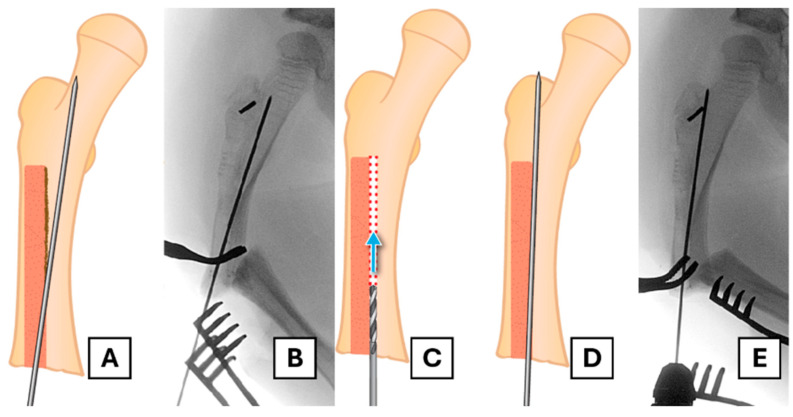
Establishing the correct path for the revision rod. (**A**,**B**) Illustration and corresponding radiograph showing the guidewire’s trajectory being deflected medially by the sclerotic pseudocortex of the old rod tract. (**C**) A drill is used to create a new pathway through the blocking bone. (**D**,**E**) Illustration and radiograph confirming the correct trajectory of the guidewire towards the greater trochanter apex.

Once successful removal is completed, the placement of a revised FD rod is undertaken in a standard open osteotomy fashion. There may be a pseudocortex present in the proximal segment, dividing the previous rod tract from the desired line of exit through the greater trochanter. Accurate placement of the guidewire can be challenging, as it tends to follow the previously created channel and may be pushed medially by the sclerotic edge (Figure 5A,B). To solve this problem, curettage or drilling may be necessary to allow the guidewire to follow the appropriate path through the proximal segment to its exit point. We used a 2 mm drill bit to create a pathway in the desired line, creating access for the guidewire (Figure 5C–E).

The revision rod can then be implanted in a standard fashion. Deformity should be corrected at the fracture site. Stability of the fracture site and/or the osteotomy site should be evaluated. If there is any doubt about stability, supplemental plate fixation should be considered. In this case, however, the stability was adequate, and we did not add a plate fixation. We placed demineralized bone matrix mixed with removed callus and cancellous bone back to the osteotomy site. The distal male part should be centered in the epiphysis, as a study has shown that its position can affect the pull-out rate and revision rate [6]. However, due to the operative time, blood loss, and risk of instability with an additional osteotomy, we felt that it would pose more risks than benefits; therefore, we did not perform an additional osteotomy and accepted the position.

### 4.5. Closure and Post-Operative Care

Thorough irrigation of the surgical wound should be performed prior to bone grafting. The fascia is closed with interrupted size 0 absorbable sutures. The remaining layers are closed with respect to the original anatomy. Interrupted size 2.0 absorbable sutures are placed in the subcutaneous tissue, and a size 4.0 rapidly absorbable suture is used for subcuticular closure. Wound closure strips are placed, and gauze is used to cover the wound. A three-sided slab covering from the ankle to the ASIS area, limiting hip abduction, was applied.

**Figure 6:**
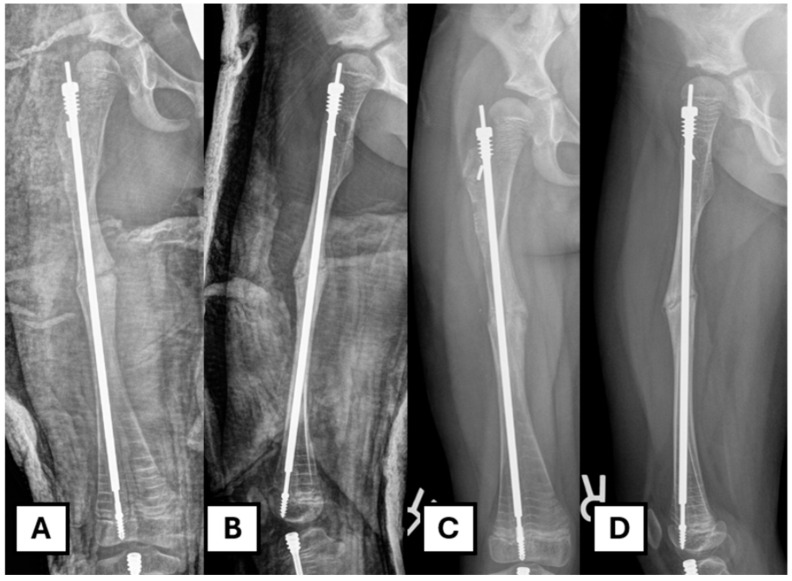
Post-operative image after revision. (**A**,**B**) Immediate post-operative radiograph. (**C**,**D**) Six weeks post-operative radiograph showing progression of osteotomy site healing.

The patient is admitted overnight for pain control. Two doses of post-operative prophylactic antibiotics are administered. A standard AP and lateral X-ray of the operated femur is obtained on day one post-op. The patient remains non-weight-bearing on the operated leg for six weeks post-op. A follow-up in the clinic is performed two weeks after the initial visit, at which point the wound is reviewed. A decision is made regarding the progression of weight-bearing status when the X-ray shows adequate healing (Figure 6).

## 5. Discussion

We have presented our strategy for the revision of an FD rod in a femur where distal migration of the proximal fixation has occurred. To date, we have been unable to identify any discussion in the literature regarding options in this specific scenario. The methods employed in this case may not be universally applicable, and the risks and benefits of different techniques should be assessed for each individual case.

In pre-operative planning, we considered several approaches to retrieval. Retrograde retrieval of the rod is possible if a guidewire can be passed into the rod from a proximal entry point; however, reaming over the guidewire would likely result in significant bone loss around the greater trochanter, sacrificing the potential remaining bone for revision fixation (Figure 1). We could consider leaving the existing rod in situ and bypassing it with a second rod, but it may prove challenging and would likely complicate any future exchange. Creating a clamshell osteotomy or another osteotomy at the level of the proximal fixation could allow for retrograde extraction, but the technique would involve more significant soft tissue stripping or the creation of a second fracture site. Lastly, although a retrograde telescopic nail technique has recently shown a promising result and might be another choice for revision [7]. We prefer not to use the method as it entails performing arthrotomy of the knee, which we would like to avoid.

Our rationale for recommending the retrograde trephine approach is that priority should be given to preserving proximal bone stock to avoid compromising the fixation of the revision device. The bone loss with our described method is in an area that minimally affects the biomechanics of the revision rod and appears to be the best compromise when all options are considered.

The selected trephine needs to have an inner diameter larger than the female rod diameter while having an outer diameter as close as possible to that of the proximal fixation, a tool not freely available off the shelf. McClure et al. have previously discussed the utility of specially designed trephines for the retrieval of FD rods in the specific setting of congenital pseudoarthrosis of the tibia [8]. The use of custom or rod-size-specific trephines, which may need to be longer than normal, depending on the length of the female component to be traversed, would ensure minimal unnecessary bone loss and could allow for lower torque generation during the procedure. However, this could also present a limitation for this technique if the appropriate size and length of the trephines are not available. According to the FD-rod implant guide, one would need a trephine of 7.0 mm diameter for a 3.2 mm nail, and up to 11.5 mm for a 6.0 mm nail. The appropriate size of the trephine is essential, as models show that torque failure occurs at much lower levels in OI compared to controls [9]. In this case study, a combination of hand and power-driven trephines was used, as the available trephine compatible with a power driver was insufficient in length. This retrograde reaming technique would theoretically work with the humerus and tibia as well, in the case that the canal size is sufficient and one could find the appropriate size trephine.

In reference to bone stock, the type of OI and the current pharmaceutical treatment need to be taken into account. The technique described may be considered when bone stock is adequate and bone morphology allowed. This technique should be avoided in the absence of a sufficient canal, which may be seen particularly in type III OI. Bisphosphonate therapy is commonly employed in the management of patients with OI, but increasing mineral density has been shown to increase brittleness, and caution is advised in these patients when utilizing a torque-producing procedure [10].

Other than the surgical technique, early detection of rod migration is of critical importance. When there is a sign of distal migration of the female component, early revision would also help avoid the need for this technique, as the rod can be removed from the trochanteric apophysis without needing to remove too much bone stock.

## 6. Conclusions

This technique provides a rationale and a step-by-step process to address a rare and challenging surgical scenario. The priority of maintaining proximal bone stock for revision device fixation is emphasized, aiming to avoid the creation of difficult-to-salvage situations. Pre-operative sourcing of appropriate trephine options will allow for a smooth intra-operative flow. This case also highlights the importance of follow-up and timely intervention to avoid more significant complications associated with implant removal.

## Data Availability

The original contributions presented in this study are included in the article. Further inquiries can be directed to the corresponding author.

## Abbreviations

The following abbreviations are used in this manuscript:

OI	Osteogenesis Imperfecta
FD	Fassier–Duval
GT	Greater Trochanter
AP	Anteroposterior

## References

[1] Fassier F.R. (2021). Osteogenesis Imperfecta-Who Needs Rodding Surgery?. Curr. Osteoporos. Rep..

[2] Celin M.R., Kruger K.M., Caudill A., Nagamani S.C.S., Harris G.F., Smith P.A. (2020). A Multicenter Study of Intramedullary Rodding in Osteogenesis Imperfecta. JBJS Open Access.

[3] DellaPolla A., Samson K., Dalamaggas A., Strudthoff E., Esposito P., Wallace M. (2023). Long-Term Results of Initial and Reoperation Surgeries with Fassier-Duval Intramedullary Rods in Femurs and Tibias of Children with Osteogenesis Imperfecta. JBMR Plus.

[4] AbdEl-Moneem Ghaly N., Ahmed A.R., Wafa T.A. (2021). Outcomes of Using Different Generations of Telescopic Intramedullary Nails in Lower Limbs in Management of Osteogenesis Imperfecta Patients. A Systematic Review. QJM.

[5] Birke O., Davies N., Latimer M., Little D.G., Bellemore M. (2011). Experience with the Fassier-Duval telescopic rod: First 24 consecutive cases with a minimum of 1-year follow-up. J. Pediatr. Orthop..

[6] Holmes K., Gralla J., Brazell C., Carry P., Tong S., Miller N.H., Georgopoulos G. (2020). Fassier-Duval Rod Failure: Is It Related to Positioning in the Distal Epiphysis?. J. Pediatr. Orthop..

[7] Georges S., Saliba I., Finidori G., Haumont E., Pannier S., Pejin Z. (2025). Retrograde femoral nailing for deformity correction and fracture treatment in osteogenesis imperfecta: Clinical and radiological assessment of a novel technique. SICOT J..

[8] McClure P.K., Franzone J.M., Herzenberg J.E. (2022). Challenges with Fassier-Duval rod exchanges in congenital pseudarthrosis of the tibia: Explant roadblock and solution. J. Pediatr. Orthop. B.

[9] Camacho N.P., Hou L., Toledano T.R., Ilg W.A., Brayton C.F., Raggio C.L., Root L., Boskey A.L. (1999). The material basis for reduced mechanical properties in oim mice bones. J. Bone Miner. Res..

[10] Burr D.B. (2020). Fifty years of bisphosphonates: What are their mechanical effects on bone?. Bone.

